# ﻿Five new species of the *Pholcusphungiformes* species group (Araneae, Pholcidae) from South Korea

**DOI:** 10.3897/zookeys.1178.104780

**Published:** 2023-09-04

**Authors:** Chang Moon Jang, Yang Seop Bae, Sue Yeon Lee, Jung Sun Yoo, Seung Tae Kim

**Affiliations:** 1 Division of Life Sciences, College of Life Sciences and Bioengineering, Incheon National University, Incheon 22012, Republic of Korea Incheon National University Incheon Republic of Korea; 2 Life and Environment Research Institute, Konkuk University, Seoul 05029, Republic of Korea Konkuk University Seoul Republic of Korea; 3 Species Diversity Research Division, National Institute of Biological Resources, Incheon 22689, Republic of Korea National Institute of Biological Resources Incheon Republic of Korea

**Keywords:** Biodiversity, description, mixed forest, morphology, spider, taxonomy

## Abstract

Five new spider species of the genus *Pholcus* Walckenaer, 1805, *P.duryun***sp. nov.**, *P.hwaam***sp. nov.**, *P.mohang***sp. nov.**, *P.worak***sp. nov.**, and *P.yangpyeong***sp. nov.**, belonging to the *P.phungiformes* group in the family Pholcidae C. L. Koch, 1850, are newly described from South Korea. These new species were collected from mixed forests in mountainous, hilly, and coastal terrains. This study provides the diagnoses, detailed descriptions, distribution maps, and taxonomic photographs of these new species.

## ﻿Introduction

**Figure 1.**
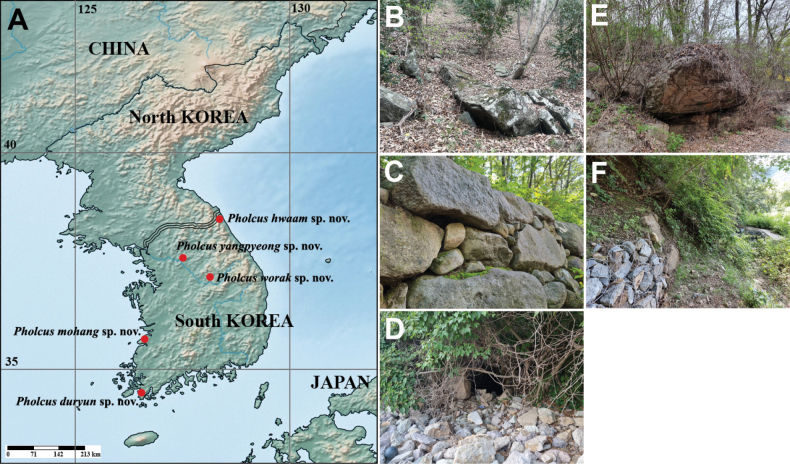
Distribution map and habitats of new *Pholcus* spiders **A** distribution of the new *Pholcus* spiders in South Korea **B** habitat of *Pholcusduryun* sp. nov. **C** habitat of *Pholcushwaam* sp. nov. **D** habitat of *Pholcusmohang* sp. nov. **E** habitat of *Pholcusworak* sp. nov. **F** habitat of *Pholcusyangpyeong* sp. nov.

*Pholcus* Walckenaer, 1805, the most diverse genus in the family Pholcidae C. L. Koch, 1850, currently includes 384 species belonging to 21 species groups (Huber 2011; Huber et al. 2018; Yao et al. 2021; WSC 2023). One of species group in the genus, *Pholcusphungiformes* species group, is largely restricted to northern and northeastern China, the Korean Peninsula, and the Far East of Russia and occurs mainly in dusky, humid spaces such as rock walls, road drains, and cave entrances in mountainous regions (Huber 2011; Kim et al. 2016; Lee et al. 2021a; Yao et al. 2021). The *P.phungiformes* species group can be distinguished from other species groups by the following combination of characters: male chelicerae usually with frontal apophyses, male palpal tibia with prolatero-ventral modification, procursus usually with dorsal spines, the male genital bulb without appendix or with pseudo-appendix arising from uncus, and a sclerotized epigynum with knob (Huber 2011; Yao et al. 2021). Taxonomic research on this group has recently been active in China and South Korea and 110 species have been recorded in the region to date; 70 from China, 39 from South Korea (*P.extumidus* Paik, 1978, is found in both South Korea and Japan), and one species from Russia (Lee et al. 2021a, b; Yao et al. 2021; Lu et al. 2022; Jang et al. 2023; Zhao et al. 2023a, b). Five new *Pholcus* spiders belonging to the *P.phungiformes* species group were collected during surveys on the spider fauna in mountainous, hilly, and coastal mixed forests during 2019–2022 (Fig. 1). The present study describes these new species. For each species we provide a diagnosis, detailed description, distribution map, and taxonomic photographs.

## ﻿Materials and methods

All specimens were collected by hands and preserved in 98% ethyl alcohol and external morphology was examined under a Leica S8APO (Singapore) stereomicroscope. Images were captured with a Dhyana 400DC zoom digital camera (China) mounted on a Leica S8APO and assembled using Helicon Focus 8.2.0 image stacking software (Khmelik et al. 2006). Measurements of body parts were made with an ocular micrometer and are recorded in millimeters. Internal genitalia of females were removed and treated in 10% KOH for 2 h before illustration. Leg measurements are shown as: total length (femur, patella, tibia, metatarsus, tarsus). Morphological terminology follows Huber (2011). All type specimens studied are deposited in the National Institute of Biological Resources, Incheon, South Korea (**NIBR**). The distribution map was produced by modifying SimpleMappr (Shorthouse 2010). The following abbreviations are used in the descriptions: **ALE** = anterior lateral eye, **AME** = anterior median eye, **PLE** = posterior lateral eye; **PME** = posterior median eye, **ALE-AME** = distance between ALE-AME, **ALE-PLE** = distance between ALE-PLE, **AME-AME** = distance between AMEs, **AME-PME** = distance between AME-PME, **PLE-PME** = distance between PLE-PME, **PME-PME** = distance between PMEs in the eye region; **L/d** = length/diameter in the leg measurement.

## ﻿Taxonomic account

### ﻿Family Pholcidae C. L. Koch, 1850

**Subfamily Pholcinae C.L. Koch, 1850**

#### ﻿Genus Pholcus

Taxon classificationAnimalia

Walckenaer, 1805

2E5C690A-7A25-5939-B7E8-B081FC762B1C

##### Diagnosis and detailed description.

See Huber (2011).

##### Type species.

*Araneaphalangioides* Fuesslin, 1775.

###### ﻿*Pholcusphungiformes* species group

**Diagnosis and description.** See Huber (2011) and Yao et al. (2021).

#### ﻿ Pholcus duryun sp. nov.

Taxon classificationAnimalia

B2CA1C19-3338-568C-A5F5-8C1D052C41D0

https://zoobank.org/83A070B1-B32E-4D63-B6CC-8CC1685F9B91

Figs 2 , 8A

##### Material examined.

***Holotype***: South Korea • ♂; Jeollanam-do, Haenam-gun, Samsan-myeon, Daeheungsa-gil, Mt. Duryunsan; 34°29.6'N, 126°37.0'E, 88 m; 7 August 2019; S.T. Kim & S.Y. Lee leg.; NIBR #NUHGIV0000000001. ***Paratypes***: South Korea • 2♂♂ and 6♀♀ same data as for holotype; NIBR #NUHGIV0000000004–11.

##### Diagnosis.

**Figure 2.**
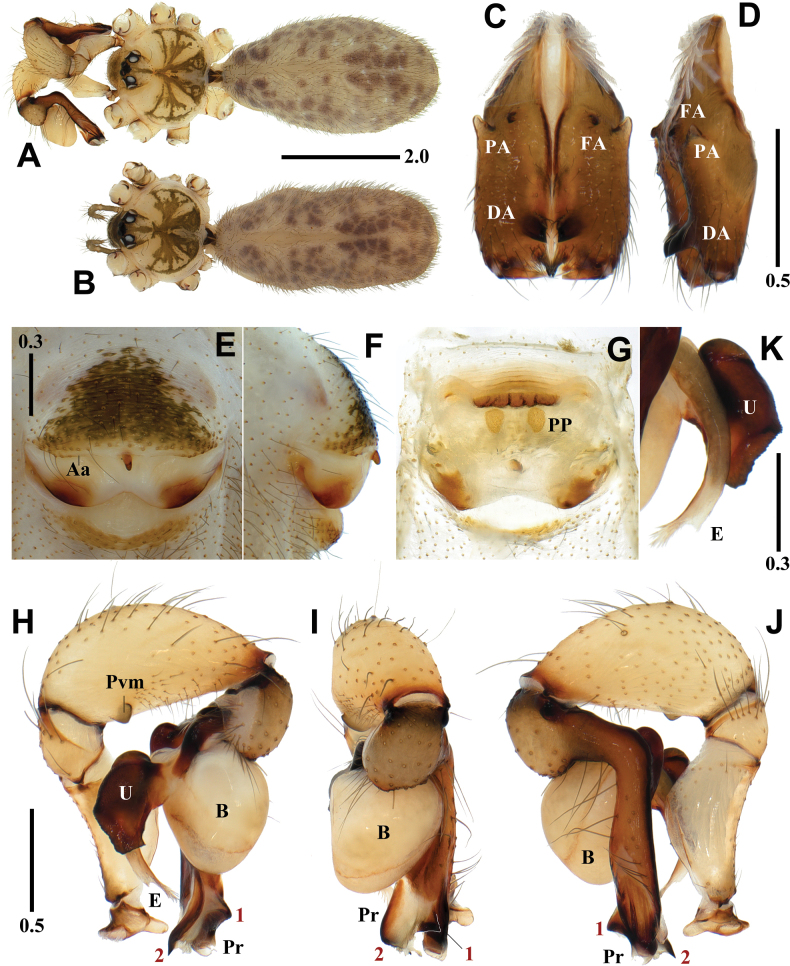
*Pholcusduryun* sp. nov., holotype male (**A, C, D, H–K**) and paratype female (**B, E–G**) **A, B** habitus **C, D** chelicerae (**C** frontal view **D** lateral view) **E, F** epigynum (**E** ventral view **F** lateral view) **G** internal genitalia, dorsal view **H–J** palp (1 = prolateral apophysis, 2 = ventrodistal apophysis **H** prolateral view **I** frontal view **J** retrolateral view) **K** bulbal processes. Abbreviations: Aa = anterior arch of epigynum, B = bulb, DA = distal apophysis, E = embolus, FA = frontal apophysis, PA = proximo-lateral apophysis, PP = pore plate, Pr = procursus, Pvm = prolatero-ventral modification, U = uncus. Scale bars in mm.

*Pholcusduryun* sp. nov. is similar to *P.extumidus* Paik, 1978 in the shape of the genital organ and body appearance but can be easily distinguished from the latter by the combination of the following characters: male - trochanter with short retrolatero-ventral apophysis; palpal tibia with quadrangular prolatero-ventral modification (Fig. 2H); uncus with rather smooth edge (Fig. 2H); procursus with triangular prolateral apophysis (numbered 1 in Fig. 2H–J) and slightly curved ventrodistal apophysis (numbered 2 in Fig. 2H–J) vs trochanter with long retrolatero-ventral apophysis; palpal tibia with semicircular prolatero-ventral modification; uncus with distinctive serrated edge; procursus with claw-shaped prolateral apophysis and strongly curved ventrodistal apophysis in *P.extumidus* (Paik 1978: 123, figs 47–49). Female - epigynum with straight anterior arch, median portion narrowly depressed postero-medially, and pore plates longitudinal (Fig. 2E) vs epigynum with recurved anterior arch, median portion broadly depressed postero-medially, and pore plates slanted in *P.extumidus* (Paik 1978: 123, figs 52–54).

##### Description.

**Male** (holotype). Habitus as in Fig. 2A. Total length 5.60. Carapace: 1.74 long/1.79 wide. Eyes: AER 0.62, PER 0.68, ALE 0.16, AME 0.12, PLE 0.17, PME 0.15, ALE-PLE contiguous, ALE-AME 0.05, AME-AME 0.04, AME-PME 0.06, PME-PLE 0.04, PME-PME 0.24. Chelicera: 0.95 long/0.27 wide. Endite: 0.51 long/0.34 wide. Labium: 0.32 long/0.39 wide. Sternum: 0.80 long/1.15 wide. Legs: I 44.27 (11.38, 0.64, 11.43, 18.43, 2.39), II 29.80 (8.18, 0.69, 7.55, 11.83, 1.55), III 20.68 (6.11, 0.59, 5.08, 7.75, 1.15), IV 27.15 (7.91, 0.65, 6.90, 10.29, 1.40), tibia I L/d 60. Palp: 4.05 (0.80, 0.38, 1.27, -, 1.60). Abdomen: 3.65 long/1.92 wide.

Carapace pale yellowish brown, cephalic region with a pale blackish brown median band, thoracic region with pale blackish brown radial and marginal bands (Fig. 2A). Chelicera with three apophyses; blunt proximo-lateral apophysis slightly protruding diagonally upward out of chelicera, small and blunt frontal apophysis protruding forward, and thick and pointed distal apophysis slightly protruding diagonally downward (Fig. 2C, D). Legs yellowish brown, retrolateral trichobothrium on tibia I at 8% proximally, tarsus I with 30 pseudosegments, femora, tibiae, and metatarsi with one or two pale blackish brown proximal and distal annuli, leg formula I-II-IV-III.

Abdomen elliptical, pale grayish brown with a long cardiac pattern and many blackish brown irregular spots (Fig. 2A). Palp (Fig. 2H–K): trochanter with blunt finger-shaped retrolatero-ventral apophysis, much shorter than femur; palpal tibia with a quadrangular prolatero-ventral modification (Fig. 2H); bulb pale yellowish brown, pocket-shaped; uncus dark blackish brown and almost rectangular with round and truncated sides having fine scales, edge rather smooth, pseudo-appendix absent; embolus weakly sclerotized and cut off-shaped with some semitransparent fringed distal processes, thick and long, curved (Fig. 2H, K); procursus large and long, brown with blackish brown margin, large ventral knee roundly swollen and strongly curved, two distal apophyses present, prolateral apophysis triangular with a pointed tip (numbered 1 in Fig. 2H–J) and ventrodistal apophysis long with a pointed tip (numbered 2 in Fig. 2H–J), one thin and short dorsal spine present (Fig. 8A).

**Female** (paratype). General appearance similar to male, habitus as in Fig. 2B. Total length 5.72. Carapace: 1.69 long/1.74 wide. Eyes: AER 0.60, PER 0.67, ALE 0.13, AME 0.12, PLE 0.17, PME 0.14, ALE-PLE contiguous, ALE-AME 0.04, AME-AME 0.04, AME-PME 0.06, PME-PLE 0.04, PME-PME 0.22. Chelicera: 0.83 long/0.27 wide. Endite: 0.55 long/0.31 wide. Labium: 0.30 long/0.37 wide. Sternum: 0.83 long/1.03 wide. Legs: I 34.95 (8.65, 0.65, 8.81, 14.52, 2.32), II 24.17 (6.57, 0.66, 6.03, 9.38, 1.53), III 17.67 (5.11, 0.61, 4.24, 6.58, 1.13), IV 23.80 (6.67, 0.63, 5.89, 9.19, 1.42), tibia I L/d 42. Palp: 1.27 (0.39, 0.18, 0.24, -, 0.46). Abdomen: 3.74 long/1.72 wide. Epigynum: 0.95 wide.

Legs yellowish brown, femora, tibiae, and metatarsi with one or two pale blackish brown proximal and distal annuli, leg formula I-II-IV-III. Epigynum (Fig. 2E, F): sclerotized, anterior epigynal plate strongly protruding, anterior arch with median portion straight, anterior epigynal plate and posterior epigynal plate far apart, both sides of median portion sclerotized postero-ventrally; small and short knob with a blunt tip. Internal genitalia (Fig. 2G): pore plates roundly triangular, longitudinal, and moderately far apart from each other.

##### Variation.

Tibia I in two paratype males (NIBR #NUHGIV0000000004–05): 11.24, missing. Tibia I in other five paratype females (NIBR #NUHGIV0000000007–11): 8.66 ± 0.44 (8.84, 8.83, 9.21, 8.10, 8.34).

##### Habitat.

Rock walls and under rocks in a mountainous mixed forest (Fig. 1B).

##### Distribution.

South Korea (Mt. Duryunsan, Jeollanam-do) (Fig. 1A).

##### Etymology.

The specific name is a noun in apposition referring to the type locality, Mt. Duryunsan.

#### ﻿ Pholcus hwaam sp. nov.

Taxon classificationAnimalia

FCEAB6D9-6413-5264-8BD6-461A1A1AA028

https://zoobank.org/DB85CA7B-9091-4878-837B-6055000EE138

Figs 3 , 8B

##### Material examined.

***Holotype***: South Korea • ♂; Gangwon-do, Goseong-gun, Toseong-myeon, Sinpyeong-ri, Hwaamsa Temple; 38°13.6'N, 128°28.4'E, 305 m; 21 September 2022; C.M. Jang & S.T. Kim leg.; NIBR #NUHGIV0000000002. ***Paratypes***: South Korea • 5♂♂ and 5♀♀ same data as for holotype; NIBR #NUHGIV00000000012–21.

##### Additional material examined.

*Pholcusseorakensis* Seo, 2018: South Korea • 2♂♂ 3♀♀; Gangwon-do, Inje-gun, Buk-myeon, Baekdam-ro; 38°11.0'N, 128°21.4'E, 399 m (from type locality); 21 September 2022; C.M. Jang & S.T. Kim leg.

##### Diagnosis.

**Figure 3.**
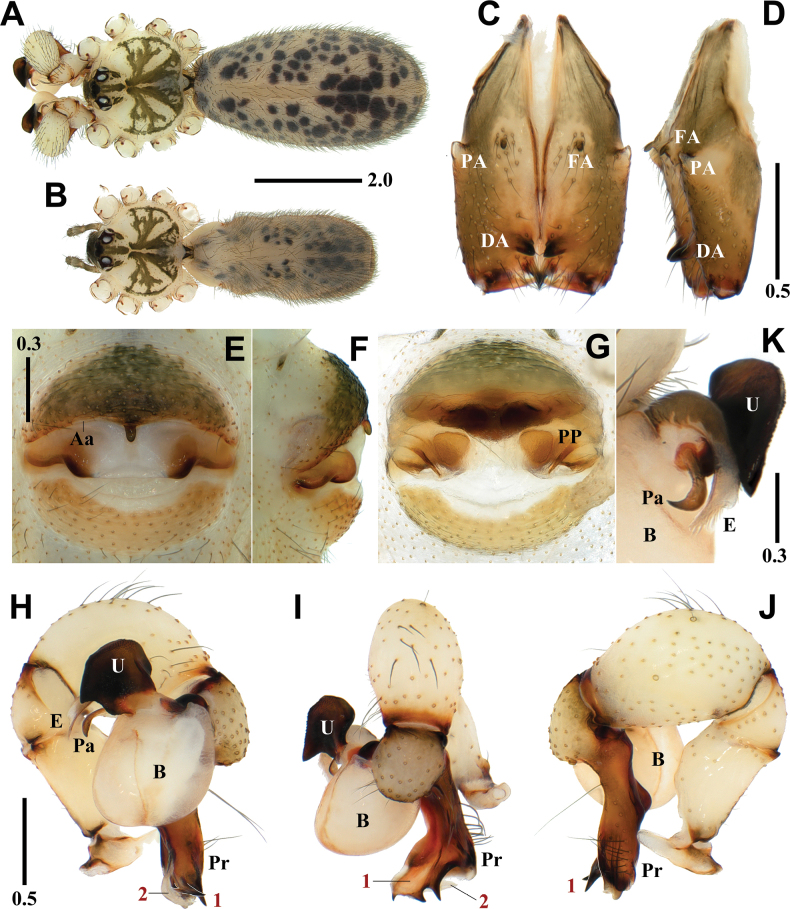
*Pholcushwaam* sp. nov., holotype male (**A, C, D, H–K**) and paratype female (**B, E–G**) **A, B** habitus **C, D** chelicerae (**C** frontal view **D** lateral view) **E, F** epigynum (**E** ventral view **F** lateral view) **G** internal genitalia, dorsal view **H–J** palp (1 = dorsodistal apophysis, 2 = ventrodistal apophysis **H** prolateral view **I** frontal view **J** retrolateral view) **K** bulbal processes. Abbreviations: Aa = anterior arch of epigynum, B = bulb, DA = distal apophysis, E = embolus, FA = frontal apophysis, PA = proximo-lateral apophysis, Pa = pseudo-appendix, PP = pore plate, Pr = procursus, U = uncus. Scale bars in mm.

**Figure 4.**
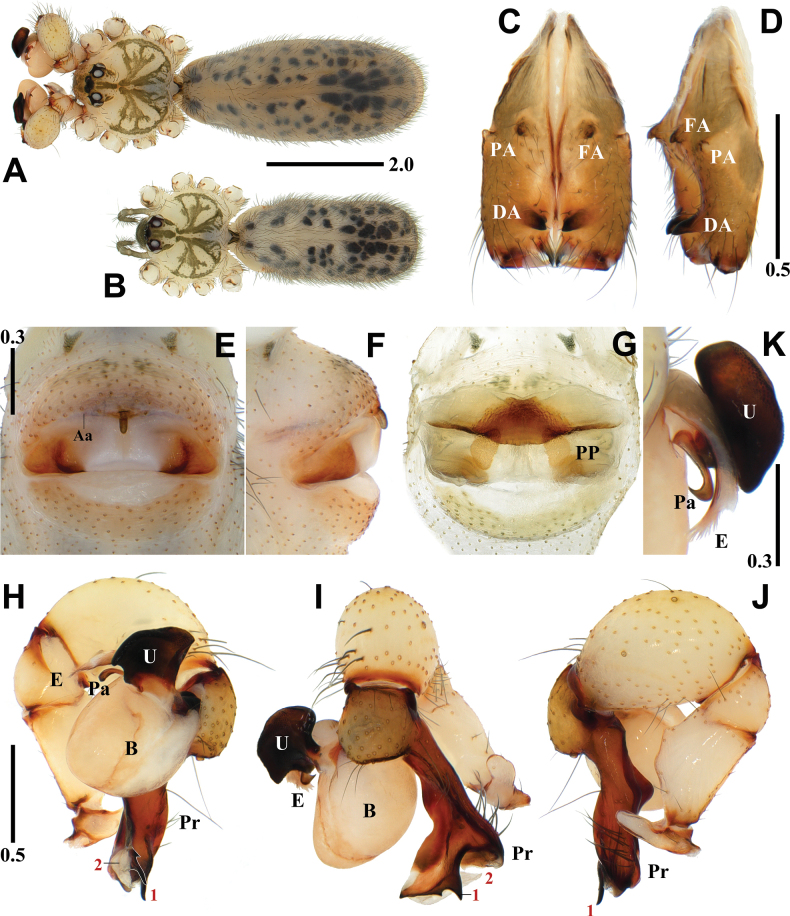
*Pholcusseorakensis* Seo, 2018, male (**A, C, D, H–K**) and female (**B, E–G**) **A, B** habitus **C, D** chelicerae (**C** frontal view **D** lateral view) **E, F** epigynum (**E** ventral view **F** lateral view) **G** internal genitalia, dorsal view **H–J** palp (1 = dorsodistal apophysis, 2 = ventrodistal apophysis **H** prolateral view **I** frontal view **J** retrolateral view) **K** bulbal processes. Abbreviations: Aa = anterior arch of epigynum, B = bulb, DA = distal apophysis, E = embolus, FA = frontal apophysis, PA = proximo-lateral apophysis, Pa = pseudo-appendix, PP = pore plate, Pr = procursus, U = uncus. Scale bars in mm.

*Pholcushwaam* sp. nov. is similar to *P.seorakensis* Seo, 2018 in the shape of the genital organ and body appearance but can be easily distinguished from the latter by the combination of the following characters: male - uncus rectangular with an angular side (Fig. 3H); procursus with a broad dorsodistal apophysis having one pointed tip and two large and small separated tips in prolateral view (numbered 1 in Fig. 3H–J) vs uncus rectangular with two angular sides; procursus with broad dorsodistal apophysis having one pointed tip and two large and small connected tips in prolateral view in *P.seorakensis* (numbered 1 in Fig. 4H–J; Seo 2018: 256, figs 3G–J). Female - median portion depressed postero-laterally with triangular pore plates (Fig. 3G) vs median portion undepressed with triangular pore plates in *P.seorakensis* (Fig. 4E–G; Seo 2018: 256, fig. 3L).

##### Description.

**Male** (holotype). Habitus as in Fig. 3A. Total length 5.59. Carapace: 1.69 long/1.86 wide. Eyes: AER 0.78, PER 0.87, ALE 0.20, AME 0.16, PLE 0.22, PME 0.20, ALE-PLE contiguous, ALE-AME 0.05, AME-AME 0.07, AME-PME 0.07, PME-PLE 0.05, PME-PME 0.31. Chelicera: 1.13 long/0.35 wide. Endite: 0.57 long/0.35 wide. Labium: 0.32 long/0.37 wide. Sternum: 0.86 long/1.24 wide. Legs: I 50.59 (13.00, 0.76, 13.03, 21.36, 2.44), II 34.77 (9.63, 0.70, 8.84, 14.37, 1.23), III 22.16 (6.47, 0.61, 5.49, 8.40, 1.19), IV 30.59 (8.78, 0.70, 7.74, 11.99, 1.38), tibia I L/d 76. Palp: 2.98 (0.60, 0.29, 0.96, -, 1.13). Abdomen: 3.90 long/1.95 wide.

Carapace pale yellowish brown, cephalic region with pale blackish brown median and marginal bands, thoracic region with pale blackish brown radial and marginal bands (Fig. 3A). Chelicera with three apophyses; blunt proximo-lateral apophysis slightly protruding diagonally upward out of chelicera, small and blunt frontal apophysis protruding forward, and thick and pointed distal apophysis slightly protruding diagonally downward (Fig. 3C, D). Legs yellowish brown, retrolateral trichobothrium on tibia I at 6% proximally, tarsus I with 30 pseudosegments, femora, tibiae, and metatarsi with one or two pale to dark blackish brown proximal and distal annuli, leg formula I-II-IV-III. Abdomen elliptical, turbid gray with a long cardiac pattern and many black irregular spots (Fig. 3A). Palp (Fig. 3H–K): trochanter with blunt and finger-like retrolatero-ventral apophysis, shorter than femur; palpal tibia with finger-shaped prolatero-ventral modification hidden by uncus; bulb pale yellowish brown, pocket-shaped; uncus dark blackish brown and square with rounded edge having fine scales, angled on one side, edge finely serrated, pseudo-appendix thick and long, claw-shaped (Fig. 3H, K); embolus thick and weakly sclerotized with some semitransparent fringed distal processes and oblique broad tip, slightly curved (Fig. 3H, K); procursus large and long, brown with blackish brown margin, small ventral knee roundly swollen and straight, two distal apophyses present, dorsodistal apophysis broad with three pointed tips (numbered 1 in Fig. 3H–J) and white ventrodistal apophysis strongly curved with a pointed tip (numbered 2 in Fig. 3H, I), one thick and long dorsal spine present (Fig. 8B).

**Female** (paratype). General appearance similar to male, habitus as in Fig. 3B. Total length 4.66. Carapace: 1.62 long/1.74 wide. Eyes: AER 0.74, PER 0.81, ALE 0.19, AME 0.15, PLE 0.20, PME 0.19, ALE-PLE contiguous, ALE-AME 0.06, AME-AME 0.05, AME-PME 0.08, PME-PLE 0.04, PME-PME 0.29. Chelicera: 1.04 long/0.33 wide. Endite: 0.56 long/0.32 wide. Labium: 0.30 long/0.37 wide. Sternum: 0.86 long/1.17 wide. Legs: I 37.63 (9.11, 0.71, 9.37, 15.91, 2.53), II 26.20 (6.95, 0.70, 6.55, 10.33, 1.67), III 19.27 (5.40, 0.68, 4.64, 7.26, 1.29), IV 25.42 (7.23, 0.65, 6.30, 9.69, 1.55), tibia I L/d 51. Palp: 1.56 (0.47, 0.23, 0.31, -, 0.55). Abdomen: 3.04 long/1.41 wide. Epigynum: 0.96 wide.

Legs yellowish brown, femora, tibiae, and metatarsi with one or two pale blackish brown proximal and distal annuli, leg formula I-II-IV-III. Epigynum (Fig. 3E, F): sclerotized, anterior epigynal plate strongly protruding, anterior arch with median portion slightly recurved, anterior epigynal plate and posterior epigynal plate far apart, median portion sclerotized and depressed postero-laterally; small and short knob with a blunt tip. Internal genitalia (Fig. 3G): pore plates triangular and far apart from each other.

##### Variation.

Tibia I in five paratype males (NIBR #NUHGIV00000000012–16): 12.01 ± 1.18 (13.14, 11.83, 12.64, 10.43, missing). Tibia I in other four paratype females (NIBR #NUHGIV00000000018–21): 8.63 ± 0.47 (8.29, 8.55, 9.32, 8.36).

##### Habitat.

Rock walls and under rocks in a mountainous mixed forest (Fig. 1C).

##### Distribution.

South Korea (Hwaamsa Temple, Gangwon-do) (Fig. 1A).

##### Etymology.

The specific name is a noun in apposition referring to the type locality, Hwaamsa Temple.

#### ﻿ Pholcus mohang sp. nov.

Taxon classificationAnimalia

DEAA58C1-1ACE-5A9D-AFBD-B74F5A8F7B38

https://zoobank.org/0943B5E1-4229-4255-A893-B1BE6B62278E

Figs 5 , 8C

##### Material examined.

***Holotype***: South Korea • ♂; Jeollabuk-do, Buan-gun, Byeonsan-myeon, Docheong-ri, Mohang Beach; 35°35.0'N, 126°30.3'E, 14 m; 20 July 2022; C.M. Jang & S.T. Kim leg.; NIBR #WGJTIV0000000568. ***Paratypes***: South Korea • 2♂♂ and 8♀♀ same data as for holotype; NIBR #NUHGIV00000000022–31.

##### Diagnosis.

**Figure 5.**
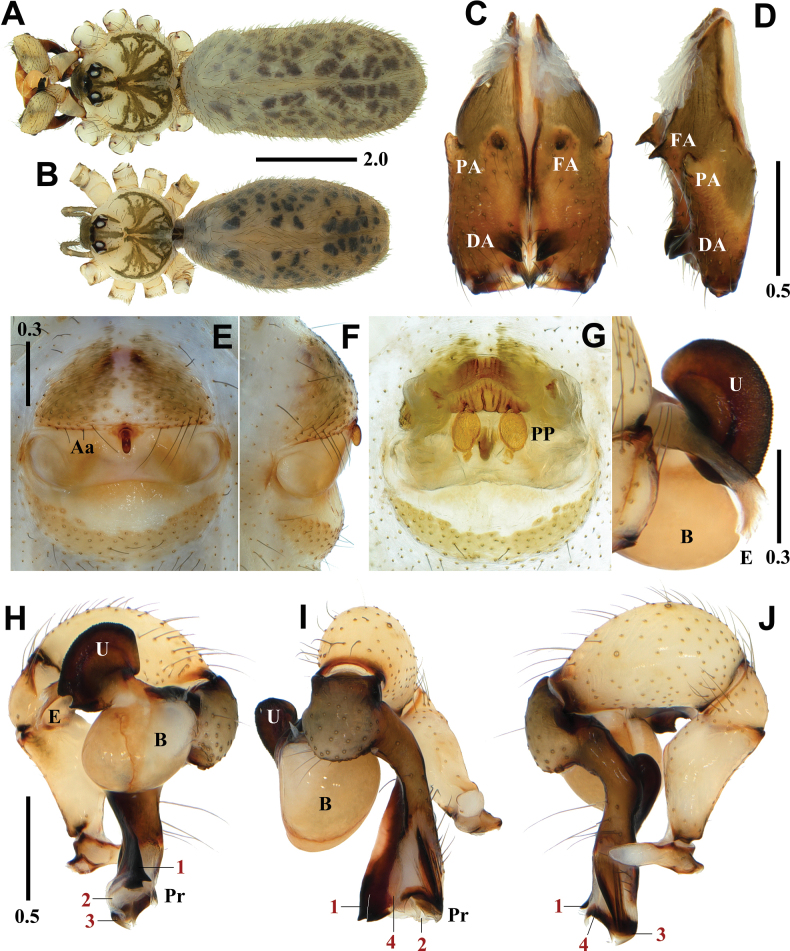
*Pholcusmohang* sp. nov., holotype male (**A, C, D, H–K**) and paratype female (**B, E–G**) **A, B** habitus **C, D** chelicerae (**C** frontal view **D** lateral view) **E, F** epigynum (**E** ventral view **F** lateral view) **G** internal genitalia, dorsal view **H–J** palp (1 = prolateral apophysis, 2 = ventral membranous process, 3 = ventrodistal apophysis, 4 = dorsodistal apophysis **H** prolateral view **I** frontal view **J** retrolateral view) **K** bulbal processes. Abbreviations: Aa = anterior arch of epigynum, B = bulb, DA = distal apophysis, E = embolus, FA = frontal apophysis, PA = proximo-lateral apophysis, PP = pore plate, Pr = procursus, U = uncus. Scale bars in mm.

*Pholcusmohang* sp. nov. can be easily distinguished from the other species within the *P.phungiformes* species group by the combination of the following characters: male - uncus elliptical, roundly depressed on one side and truncated on the other side (Fig. 5H); procursus with pointed prolateral apophysis (numbered 1 in Fig. 5H–J), white and slightly curved ventral membranous process (numbered 2 in Fig. 5H, I), claw-shaped ventrodistal apophysis (numbered 3 in Fig. 5H, J), and roundly depressed dorsodistal apophysis with pointed tips at both ends (numbered 4 in Fig. 5I, J). Female - anterior arch procurved with oval pore plates bordering the arch (Fig. 5G).

##### Description.

**Male** (holotype). Habitus as in Fig. 5A. Total length 5.72. Carapace: 1.77 long/1.75 wide. Eyes: AER 0.68, PER 0.73, ALE 0.17, AME 0.12, PLE 0.18, PME 0.15, ALE-PLE contiguous, ALE-AME 0.06, AME-AME 0.08, AME-PME 0.08, PME-PLE 0.05, PME-PME 0.28. Chelicera: 1.19 long/0.34 wide. Endite: 0.53 long/0.36 wide. Labium: 0.22 long/0.38 wide. Sternum: 0.86 long/1.20 wide. Legs: I 49.40 (12.65, 0.69, 12.73, 21.18, 2.15), II 34.05 (9.41, 0.69, 8.77, 13.70, 1.48), III 21.23 (6.21, 0.60, 5.19, 8.17, 1.06), IV 28.76 (8.41, 0.57, 7.22, 11.33, 1.23). Palp: 3.56 (0.72, 0.35, 1.03, -, 1.46). Abdomen: 3.95 long/1.88 wide.

Carapace pale yellowish brown, cephalic region with pale blackish brown median and marginal bands, thoracic region with pale blackish brown radial and marginal bands (Fig. 5A). Chelicera with three apophyses; blunt proximo-lateral apophysis protruding diagonally upward out of chelicera, small and pointed frontal apophysis slightly protruding downward, and thick and pointed distal apophysis slightly protruding diagonally downward (Fig. 5C, D). Legs yellowish brown, retrolateral trichobothrium on tibia I at 6% proximally, tarsus I with 26 pseudosegments, femora, tibiae, and metatarsi with one or two pale to dark blackish brown proximal and distal annuli, leg formula I-II-IV-III. Abdomen elliptical, turbid gray with a long cardiac pattern and many black irregular spots (Fig. 5A). Palp (Fig. 5H–K): trochanter with blunt and finger-shaped retrolatero-ventral apophysis, shorter than femur; palpal tibia with finger-shaped prolatero-ventral modification hidden by uncus; bulb pale yellowish brown, pocket-shaped; uncus dark blackish brown and semicircular with a rounded edge having fine scales, roundly depressed on one side and truncated on the other side, edge finely serrated, pseudo-appendix absent (Fig. 5H); embolus thick and weakly sclerotized with some semitransparent fringed distal processes and oblique broad tip, slightly curved (Fig. 5H, K); procursus large and long, brown with blackish brown margin, large ventral knee roundly swollen and curved, three apophyses and one process present, prolateral apophysis pointed (numbered 1 in Fig. 5H–J), ventral membranous process white and slightly curved (numbered 2 in Fig. 5H, I), ventrodistal apophysis claw-shaped (numbered 3 in Fig. 5H, J), dorsodistal apophysis broad and roundly depressed with pointed tips at both ends (numbered 4 in Fig. 5I, J), one thin and short dorsal spine present (Fig. 8C).

**Female** (paratype). General appearance similar to male, habitus as in Fig. 5B. Total length 4.90. Carapace: 1.56 long/1.58 wide. Eyes: AER 0.62, PER 0.66, ALE 0.16, AME 0.10, PLE 0.15, PME 0.14, ALE-PLE contiguous, ALE-AME 0.05, AME-AME 0.07, AME-PME 0.05, PME-PLE 0.04, PME-PME 0.26. Chelicera: 1.01 long/0.32 wide. Endite: 0.48 long/0.27 wide. Labium: 0.29 long/0.37 wide. Sternum: 0.79 long/1.08 wide. Legs: I 32.33 (8.24, 0.68, 8.24, 13.23, 1.94), II 21.81 (6.08, 0.58, 5.53, 8.36, 1.26), III 16.12 (4.63, 0.57, 3.92, 6.01, 0.99), IV 22.10 (6.40, 0.65, 5.53, 8.26, 1.26). Palp: 1.18 (0.37, 0.16, 0.23, -, 0.42). Abdomen: 3.34 long/1.79 wide. Epigynum: 0.94 wide.

Legs yellowish brown, femora, tibiae, and metatarsi with one or two pale blackish brown proximal and distal annuli, leg formula I-IV-II-III. Epigynum (Fig. 5E, F): sclerotized, anterior epigynal plate strongly protruding, anterior arch with median portion almost straight, anterior epigynal plate and posterior epigynal plate far apart; both sides of median portion unsclerotized and slightly depressed, small and short knob with a blunt tip. Internal genitalia (Fig. 5G): pore plates oval bordering the arch and moderately far apart from each other.

##### Variation.

Tibia I in two paratype males (NIBR #NUHGIV00000000022–23): 12.21, 12.09. Tibia I in other seven paratype females (NIBR #NUHGIV00000000025–31): 8.64 ± 0.48 (9.00, 8.68, 8.84, missing, 7.80, 8.88, missing).

##### Habitat.

Rock walls at the entrance of a cave in coastal hilly mixed forest (Fig. 1D).

##### Distribution.

South Korea (Mohang Beach, Jeollabuk-do) (Fig. 1A).

##### Etymology.

The specific name is a noun in apposition referring to the type locality, Mohang Beach.

#### ﻿ Pholcus worak sp. nov.

Taxon classificationAnimalia

CD485AFC-DCE4-5986-B409-86EDD20F04DD

https://zoobank.org/4A2C751D-7BDF-4154-8E6E-691B136E74B4

Figs 6 , 8D

##### Material examined.

***Holotype***: South Korea • ♂; Chungcheongbuk-do, Jecheon-si, Hansu-myeon, Songgye-ri, Mt. Woraksan National Park; 36°51.7'N, 128°5.7'E, 295 m; 10 September 2019; S.T. Kim & S.Y. Lee leg.; NIBR ##NUHGIV0000000003. ***Paratypes***: South Korea • 4♂♂ and 5♀♀ same data as for holotype; NIBR #NUHGIV00000000032–40.

##### Diagnosis.

**Figure 6.**
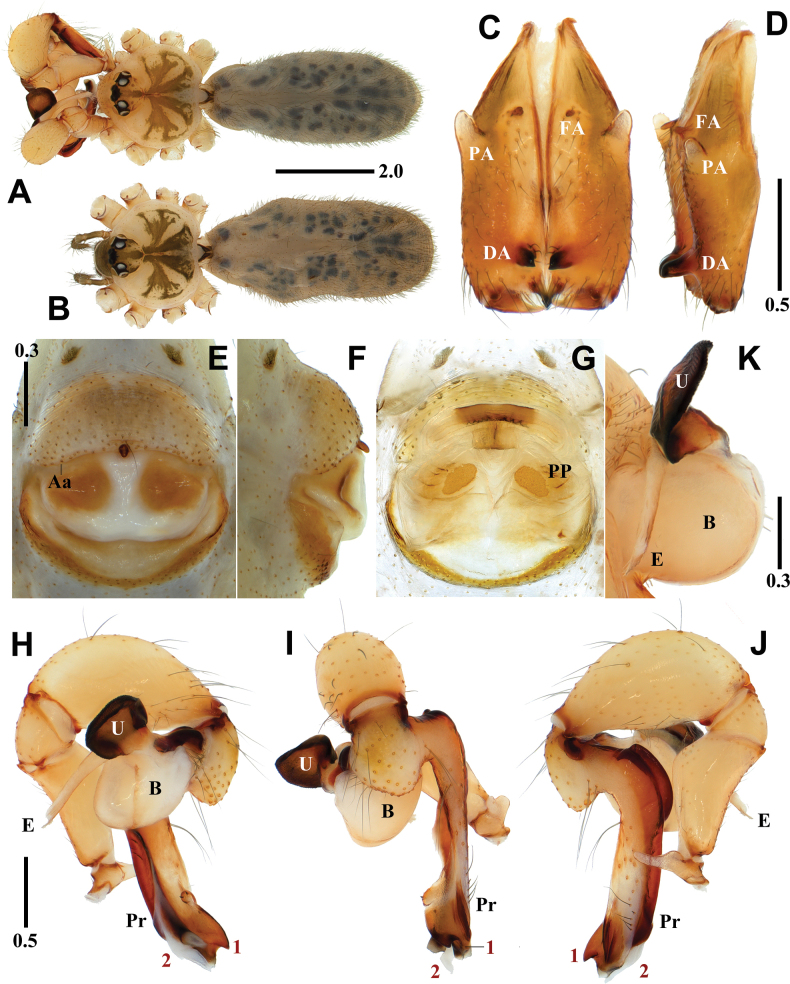
*Pholcusworak* sp. nov., holotype male (**A, C, D, H–K**) and paratype female (**B, E–G**) **A, B** habitus **C, D** chelicerae (**C** frontal view **D** lateral view) **E, F** epigynum (**E** ventral view **F** lateral view) **G** internal genitalia, dorsal view **H–J** palp (1 = dorsodistal apophysis, 2 = ventrodistal apophysis **H** prolateral view **I** frontal view **J** retrolateral view) **K** bulbal processes. Abbreviations: Aa = anterior arch of epigynum, B = bulb, DA = distal apophysis, E = embolus, FA = frontal apophysis, PA = proximo-lateral apophysis, PP = pore plate, Pr = procursus, U = uncus. Scale bars in mm.

*Pholcusworak* sp. nov. can be easily distinguished from the other species within the *P.phungiformes* species group by the combination of the following characters: male - embolus slender, conspicuously long, and straight (Fig. 6H, K); procursus with large and broadly swollen ventral knee, roundly depressed dorsodistal apophysis with pointed tip (numbered 1 in Fig. 6H–J), and long membranous ventrodistal apophysis with pointed tip (numbered 2 in Fig. 6H–J). Female - epigynum with a pair of sclerotized fried egg-shaped protuberances in median portion, pore plates elliptical, slanted, and far apart from each other in internal genitalia (Fig. 6G).

##### Description.

**Male** (holotype). Habitus as in Fig. 6A. Total length 5.17. Carapace: 1.72 long/1.75 wide. Eyes: AER 0.81, PER 0.86, ALE 0.20, AME 0.14, PLE 0.20, PME 0.18, ALE-PLE contiguous, ALE-AME 0.07, AME-AME 0.08, AME-PME 0.07, PME-PLE 0.05, PME-PME 0.31. Chelicera: 1.17 long/0.34 wide. Endite: 0.58 long/0.39 wide. Labium: 0.31 long/0.38 wide. Sternum: 0.87 long/1.15 wide. Legs: I 43.79 (11.03, 0.77, 11.22, 18.29, 2.48), II 29.24 (8.09, 0.59, 7.41, 11.69, 1.46), III 21.04 (6.12, 0.68, 5.10, 7.90, 1.24), IV 27.83 (8.11, 0.61, 6.97, 10.67, 1.47), tibia I L/d 58. Palp: 4.37 (0.80, 0.36, 1.31, -, 1.90). Abdomen: 3.45 long/1.52 wide.

Carapace pale yellowish brown, cephalic region with a pale blackish brown median band, thoracic region with pale blackish brown radial and marginal bands (Fig. 6A). Chelicera with three apophyses; blunt proximo-lateral apophysis protruding diagonally upward out of chelicera, small and blunt frontal apophysis protruding forward, and thick and pointed distal apophysis slightly protruding diagonally downward (Fig. 6C, D). Legs yellowish brown, retrolateral trichobothrium on tibia I at 5% proximally, tarsus I with 29 pseudosegments, femora, tibiae, and metatarsi with one or two pale blackish brown proximal and distal annuli, leg formula I-II-IV-III. Abdomen elliptical, turbid gray with a long cardiac pattern and many black irregular spots (Fig. 6A). Palp (Fig. 6H–K): trochanter with blunt and finger-like retrolatero-ventral apophysis, shorter than femur; palpal tibia with an eyebrow-shaped and rudimentary prolatero-ventral modification hidden by uncus; bulb pale yellowish brown, pocket-shaped, pseudo-appendix absent; uncus dark blackish brown and rectangular with a rounded edge having fine scales, edge finely serrated, pseudo-appendix absent; embolus slender and weakly sclerotized with some semitransparent fringed distal processes, conspicuously long and straight (Fig. 6H, K); procursus large and long, pale yellowish brown dorsally and brown with blackish brown margin ventrally, large ventral knee broadly swollen and smoothly curved, two distal apophyses present, dorsodistal apophysis roundly depressed with a pointed tip (numbered 1 in Fig. 6H–J) and membranous ventrodistal apophysis long with a pointed tip (numbered 2 in Fig. 6H–J), one thin and short with two short and spike like dorsal spines present on the round ridge (Fig. 8D).

**Female** (paratype). General appearance similar to male, habitus as in Fig. 6B. Total length 5.33. Carapace: 1.72 long/1.86 wide. Eyes: AER 0.75, PER 0.83, ALE 0.21, AME 0.14, PLE 0.20, PME 0.16, ALE-PLE contiguous, ALE-AME 0.04, AME-AME 0.07, AME-PME 0.09, PME-PLE 0.06, PME-PME 0.31. Chelicera: 1.08 long/0.34 wide. Endite: 0.56 long/0.32 wide. Labium: 0.33 long/0.39 wide. Sternum: 0.86 long/1.17 wide. Legs: I 39.33 (9.81, 0.70, 10.14, 16.42, 2.26), II 27.04 (7.34, 0.69, 6.91, 10.60, 1.50), III 19.96 (5.69, 0.64, 4.95, 7.51, 1.17), IV 27.20 (7.81, 0.66, 6.92, 10.29, 1.52), tibia I L/d 49. Palp: 1.62 (0.50, 0.21, 0.32, -, 0.59). Abdomen: 3.61 long/1.74 wide. Epigynum: 1.17 wide.

Legs yellowish brown, femora, tibiae, and metatarsi with one or two pale blackish brown proximal and distal annuli, leg formula I-IV≒II-III. Epigynum (Fig. 6E, F): sclerotized, anterior epigynal plate strongly protruding, anterior arch with median portion slightly curved, anterior epigynal plate and posterior epigynal plate far apart, both sides of median portion with a pair of sclerotized fried egg-shaped protuberances; small and short knob with a blunt tip. Internal genitalia (Fig. 6G): pore plates elliptical, slanted, and far apart from each other.

##### Variation.

Tibia I in four paratype males (NIBR #NUHGIV00000000032–35): 12.98 ± 0.76 (13.74, 12.48, 12.18, 13.50). Tibia I in other four paratype female (NIBR #NUHGIV00000000037–40): 9.91 ± 0.40 (10.04, 9.89, 9.38, 10.33).

##### Habitat.

Rock walls and under rocks in a mountainous mixed forest (Fig. 1E).

##### Distribution.

South Korea (Mt. Woraksan, Chungcheongbuk-do) (Fig. 1A).

##### Etymology.

The specific name is a noun in apposition referring to the type locality, Mt. Woraksan.

#### ﻿ Pholcus yangpyeong sp. nov.

Taxon classificationAnimalia

6217AE3A-F4EC-5315-8F1D-059CC0165D6E

https://zoobank.org/F8FB0A1C-2216-4500-88F2-4799F7EA7C1E

Figs 7 , 8E

##### Material examined.

***Holotype***: South Korea • ♂; Gyeonggi-do, Yangpyeong-gun, Danwol-myeon, Danwol-ro; 37°35.2'N, 127°40.7'E; alt. 275 m; 14 July 2021; C.M. Jang & S.T. Kim leg.; NIBR #WGJTIV0000000569. ***Paratypes***: South Korea • 6♂♂ and 5♀♀ same data as holotype; NIBR #NUHGIV00000000041–51.

##### Diagnosis.

**Figure 7.**
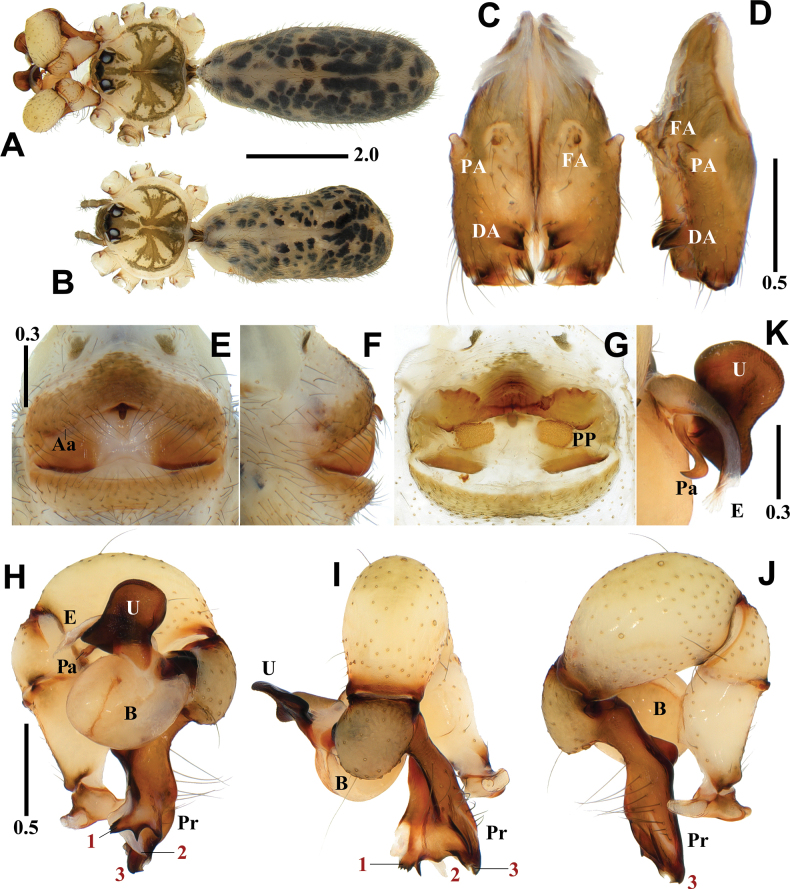
*Pholcusyangpyeong* sp. nov., holotype male (**A, C, D, H–K**) and paratype female (**B, E–G**) **A, B** habitus **C, D** chelicerae (**C** frontal view **D** lateral view) **E, F** epigynum (**E** ventral view **F** lateral view) **G** internal genitalia, dorsal view **H–J** palp (1 = prolateral apophysis, 2 = ventral membranous process, 3 = ventrodistal apophysis **H** prolateral view **I** frontal view **J** retrolateral view) **K** bulbal processes. Abbreviations: Aa = anterior arch of epigynum, B = bulb, DA = distal apophysis, E = embolus, FA = frontal apophysis, PA = proximo-lateral apophysis, Pa = pseudo-appendix, PP = pore plate, Pr = procursus, U = uncus. Scale bars in mm.

*Pholcusyangpyeong* sp. nov. can be easily distinguished from the other species within the *P.phungiformes* species group by the combination of the following characters: Male - uncus rectangular and protruding triangularly on one side; procursus with prolateral apophysis with two pointed and one serrated tips (numbered 1 in Fig. 7H, I), strongly curved ventral membranous process (numbered 2 in Fig. 7H, I), claw-shaped and slightly curved ventrodistal apophysis (numbered 3 in Fig. 7H–J). Female - anterior arch slightly recurved, pore plates rectangular and far apart from each other (Fig. 7G).

##### Description.

**Male** (holotype). Habitus as in Fig. 7A. Total length 5.27. Carapace: 1.52 long/1.68 wide. Eyes: AER 0.79, PER 0.86, ALE 0.21, AME 0.16, PLE 0.21, PME 0.18, ALE-PLE contiguous, ALE-AME 0.05, AME-AME 0.08, AME-PME 0.06, PME-PLE 0.04, PME-PME 0.32. Chelicera: 1.05 long/0.33 wide. Endite: 0.50 long/0.34 wide. Labium: 0.26 long/0.34 wide. Sternum: 0.89 long/1.07 wide. Legs: I 46.60 (11.90, 0.74, 11.87, 19.79, 2.30), II 31.16 (8.56, 0.74, 7.80, 12.51, 1.55), III 21.66 (6.31, 0.62, 5.24, 8.31, 1.18), IV 28.35 (8.35, 0.64, 7.13, 10.88, 1.35), tibia I L/d 62. Palp: 3.31 (0.56, 0.40, 1.04, -, 1.31). Abdomen: 3.75 long/1.52 wide.

Carapace pale yellowish brown, cephalic region with pale blackish brown median and marginal bands, thoracic region with pale blackish brown radial and marginal bands (Fig. 7A). Chelicera with three apophyses; blunt proximo-lateral apophysis protruding diagonally upward out of chelicera, small and pointed frontal apophysis slightly protruding downward, and thick and pointed distal apophysis slightly protruding diagonally downward (Fig. 7C, D). Legs yellowish brown, retrolateral trichobothrium on tibia I at 6% proximally, tarsus I with 30 pseudosegments, femora, tibiae, and metatarsi with one or two pale to dark blackish brown proximal and distal annuli, leg formula I-II-IV-III.

**Figure 8.**
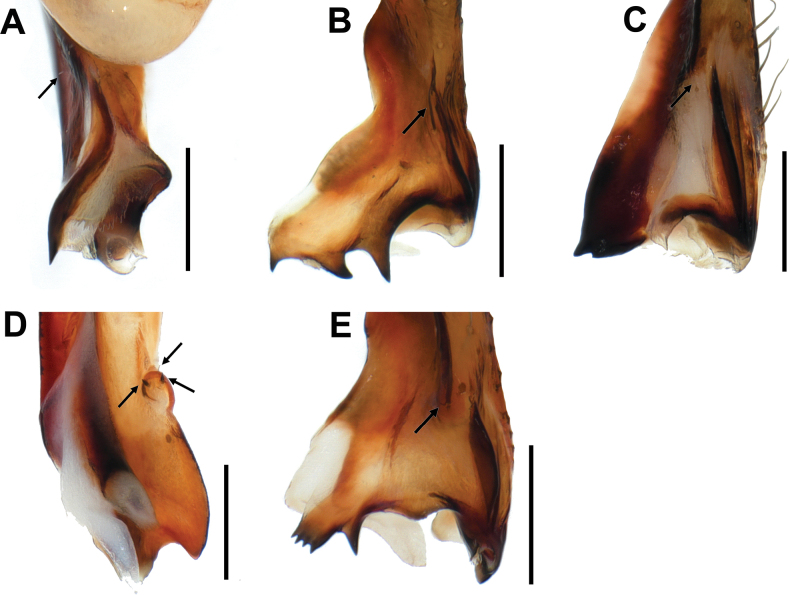
Dorsal spines of procursus **A***Pholcusduryun* sp. nov. **B***Pholcushwaam* sp. nov. **C***Pholcusmohang* sp. nov. **D***Pholcusworak* sp. nov. **E***Pholcusyangpyeong* sp. nov. (**A, D** prolateral view **B, C, E** frontal view, arrows indicate dorsal spines). Scale bars: 0.3 mm.

Abdomen elliptical, turbid gray with a long cardiac pattern and many black irregular spots (Fig. 7A). Palp (Fig. 7H–K): trochanter with blunt and finger-like retrolatero-ventral apophysis, shorter than femur; palpal tibia with a finger-shaped prolatero-ventral modification hidden by uncus; bulb pale yellowish brown, pocket-shaped; uncus dark blackish brown and rectangular with rounded edge having fine scales, protruding triangularly on one side, edge finely serrated, pseudo-appendix hook-shaped (Fig. 7H, K); embolus thick and weakly sclerotized with some semitransparent fringed distal processes and oblique broad tip, slightly curved (Fig. 7H, K); procursus large and long, brown with blackish brown margin, ventral knee roundly swollen, two apophyses and one process present, prolateral apophysis with two pointed and one serrated tips (numbered 1 in Fig. 7H, I), ventral membranous process white, membranous, and strongly curved (numbered 2 in Fig. 7H, I), ventrodistal apophysis claw-shaped and slightly curved (numbered 3 in Fig. 7H–J), one thin and recumbent dorsal spine present (Fig. 8E).

**Female** (paratype). General appearance similar to male, habitus as in Fig. 7B. Total length 4.54. Carapace: 1.53 long/1.66 wide. Eyes: AER 0.69, PER 0.76, ALE 0.19, AME 0.13, PLE 0.18, PME 0.17, ALE-PLE contiguous, ALE-AME 0.05, AME-AME 0.06, AME-PME 0.07, PME-PLE 0.05, PME-PME 0.24. Chelicera: 0.93 long/0.30 wide. Endite: 0.49 long/0.27 wide. Labium: 0.25 long/0.32 wide. Sternum: 0.82 long/1.01 wide. Legs: I 32.38 (8.16, 0.56, 8.18, 13.15, 2.33), II 21.71 (5.96, 0.58, 5.47, 8.34, 1.36), III 15.90 (4.60, 0.57, 3.74, 5.90, 1.09), IV 21.73 (6.41, 0.54, 5.46, 7.97, 1.35), tibia I L/d 49. Palp: 1.31 (0.42, 0.18, 0.24, -, 0.47). Abdomen: 3.01 long/1.36 wide. Epigynum: 1.10 wide.

Legs yellowish brown, femora, tibiae, and metatarsi with one or two pale blackish brown proximal and distal annuli, leg formula I-IV≒II-III. Epigynum (Fig. 7E, F): sclerotized and protruding anteromedially, anterior epigynal plate strongly protruding, anterior arch with median portion slightly recurved, anterior epigynal plate and posterior epigynal plate far apart, both sides of median portion sclerotized; small and short knob with a blunt tip. Internal genitalia (Fig. 7G): pore plates rectangular and far apart from each other.

##### Variation.

Tibia I in six paratype males (NIBR #NUHGIV00000000041–46): 11.38 ± 0.87 (11.61, 11.13, 12.47, 11.63, missing, 10.08). Tibia I in other four paratype female (NIBR #NUHGIV00000000048–51): 8.39 ± 0.71 (8.42, 9.13, 7.42, 8.59).

##### Habitat.

Rock walls and under rocks in mountainous mixed forest (Fig. 1F).

##### Distribution.

South Korea (Yangpyeong-gun, Gyeonggi-do) (Fig. 1A).

##### Etymology.

The specific name is a noun in apposition referred to the type locality, Yangpyeong-gun.

## Supplementary Material

XML Treatment for Pholcus

XML Treatment for Pholcus duryun

XML Treatment for Pholcus hwaam

XML Treatment for Pholcus mohang

XML Treatment for Pholcus worak

XML Treatment for Pholcus yangpyeong

## ﻿Additional information

### Author ORCIDs

Chang Moon Jang https://orcid.org/0000-0003-4343-2279

Yang Seop Bae https://orcid.org/0000-0001-7356-5633

Sue Yeon Lee https://orcid.org/0000-0002-2066-3747

Jung Sun Yoo https://orcid.org/0000-0002-3243-2006

Seung Tae Kim https://orcid.org/0000-0001-5820-9294

### Data availability

All of the data that support the findings of this study are available in the main text.
